# Health, Economic and Social Development Challenges of the COVID-19 Pandemic: Strategies for Multiple and Interconnected Issues

**DOI:** 10.3390/healthcare10050770

**Published:** 2022-04-21

**Authors:** Sigamani Panneer, Komali Kantamaneni, Udhayakumar Palaniswamy, Lekha Bhat, Robert Ramesh Babu Pushparaj, Kesavan Rajasekharan Nayar, Hilaria Soundari Manuel, F. X. Lovelina Little Flower, Louis Rice

**Affiliations:** 1 Department of Social Work, School of Social Sciences and Humanities, Central University of Tamil Nadu, Thiruvarur 610005, Tamil Nadu, India; pudhayakumar@cutn.ac.in (U.P.); lekhabhatd@gmail.com (L.B.); robertrb19@students.cutn.ac.in (R.R.B.P.); 2 Faculty of Science and Technology, University of Central Lancashire, Preston PR1 2HE, UK; 3 Global Institute of Public Health, Ananthapuri Hospitals and Research Institute, Thiruvananthapuram 695024, Kerala, India; krnayar@gmail.com; 4 Centre for Applied Research, The Gandhigram Rural Institute, Deemed to be University, Gandhigram, Dindigul 624302, Tamil Nadu, India; m.hillariasoundari@ruraluniv.ac.in; 5 Department of Social Work, Bharathiar University, Coimbatore 641046, Tamil Nadu, India; lovelina@buc.edu.in; 6 Centre for Architecture and Built Environment Research, University of the West of England, Bristol BS16 1QY, UK; louis.rice@uwe.ac.uk

**Keywords:** COVID-19, global economy, healthcare, social development, low- and middle-income countries, transdisciplinary research

## Abstract

The COVID-19-pandemic-related economic and social crises are leading to huge challenges for all spheres of human life across the globe. Various challenges highlighted by this pandemic include, but are not limited to, the need for global health cooperation and security, better crisis management, coordinated funding in public health emergencies, and access to measures related to prevention, treatment and control. This systematic review explores health, economic and social development issues in a COVID-19 pandemic context and aftermath. Accordingly, a methodology that focuses on identifying relevant literature with a focus on meta-analysis is used. A protocol with inclusion and exclusion criteria was developed, with articles from 15 December 2019 to 15 March 2022 included in the study. This was followed by a review and data analysis. The research results reveal that non-pharmaceutical measures like social distancing, lockdown and quarantine have created long-term impacts on issues such as changes in production and consumption patterns, market crashes resulting in the closure of business operations, and the slowing down of the economy. COVID-19 has exposed huge health inequalities across most countries due to social stratification and unequal distribution of wealth and/or resources. People from lower socio-economic backgrounds lack access to essential healthcare services during this critical time for both COVID-19 and other non-COVID ailments. The review shows that there is minimal literature available with evidence and empirical backup; similarly, data/studies from all countries/regions are not available. We propose that there is a need to conduct empirical research employing a trans-disciplinary approach to develop the most effective and efficient strategies to combat the pandemic and its aftermath. There is a need to explore the social and ecological determinants of this contagious infection and develop strategies for the prevention and control of COVID-19 or similar infections in future.

## 1. Introduction

This paper explores the challenges of the COVID-19 pandemic and the significance of non-pharmaceutical measures for global health and socio-economic development. The COVID-19 pandemic introduced economic and social crises that are posing huge challenges across the globe. The most serious challenges related to the COVID-19 pandemic and post-COVID future are related to the employment and incomes of millions of people, social security, income support schemes, the burden on women, the plight of migrants and informal sector workers, mental health issues, and restrictions on economic activity, including halted production, with firms unable to sell their goods and services [1]. The pandemic has also sparked fear and anxiety due to economic shocks and recession [2]. In an attempt to “flatten the curve”, various countries’ governments have imposed international border shutdowns [3,4], internal travel constraints [5] and longer periods of quarantine [6,7]. Economists have predicted that the COVID-19 pandemic will slow down Gross Domestic Product (GDP) growth by one-half a percentage point for 2020, and this applies to all countries (from 2.9% to 2.4%) [8]. Social distancing, lockdown and quarantine have high economic and social costs associated with them because they introduce changes in production and consumption patterns, which caused financial markets to crash, resulting in the closure of business operations [9]. Furthermore, this pandemic also introduced the international community to various challenges relating to global health cooperation and security, crisis management (investment in emergency preparedness) and coordinated funding during public health emergencies. The global economy has been very badly affected, especially the agro-livestock industry, hitting their lowest growth rates across various countries [10]. A decrease in inputs availability and a decrease in agricultural production during the pandemic affected food security as well [11].

Globally 3.3 billion people, which constitutes 81% of the world’s workforce, were affected by the lockdown. Of this lockdown-affected workforce, 61% were workers from the informal sector, and of this 90% were from low- and middle-income countries [12,13]. The nationwide lockdowns during the COVID-19 pandemic disunited and isolated much of the migrant populations. Due to the lack of job opportunities, millions of migrant workers were forced to return to their countries/counties/villages in a time when public transportation was closed or severely restricted. Migrants faced humanitarian and health security challenges and unusual logistical nightmares from the states where they migrated [14]. Furthermore, in many developing and underdeveloped countries, the available social security measures are weak, with a lack of access to health care and economic security [15]. As many state borders were closed, inter-country travel and trade were shut and more than 30 million people fell into poverty in the absence of active policies to protect or substitute income flows to vulnerable populations. These policies, decisions and actions severely impacted the health and wellbeing of a large section of the population [16]. With chronic low funding in rural healthcare and the economy, the pandemic has revealed the weaknesses of rural infrastructure in almost all countries [17].

## 2. Review Protocol

A systematic review has been selected for this study with exclusion and inclusion criteria applied to narrow down the literature search. A large number of academic literature and policy documents related to COVID-19 have been considered for this study. Google, Google Scholar, PubMed, Science Direct, Web of Science and Scopus were used to identify the relevant literature. Google has been used to search various policy reports and other associated documents that are not available in scientific search engines such as Science Direct, Web of Science and Scopus. This review attempted to find solutions to health, economic and social development challenges of the COVID-19 pandemic. The major objectives of the review were to understand the inter-linkages between health, economy and society, to assess the pandemic crisis, to explore health and development implications of COVID-19, to compile possible and easily workable strategies for solving problems of COVID-19, to understand the role of multi-stakeholders in time of crisis and to document innovative collaborative strategic directions to control the pandemic. Based on these objectives, the search was made to look through each database that contained the terms: COVID-19, health and development challenges, pandemics, multiple and interconnected issues, economic impact and strategies, prevention and recommendations. During the search, results from diverse sources identified some duplicate articles, especially those associated with COVID-19. Due to this, a unique combination of words was used to explore the relevant literature as follows:

COVID-19 and low- and middle-income countries
COVID-19 and developed countries
Stages of lockdown and health impacts
Lockdown and economic impact
COVID-19 and health impacts
COVID-19—disaster management
Post-pandemic context

Appropriate literature was identified from the diverse sources, based on data quality, focus area, rigourous methods, and removal of replicas; the subsequent works were scrutinised according to the exclusion and inclusion criteria listed in Table 1. This process was undertaken in different phases, with the date of publication, abstract and title considered for exclusion and inclusion. If the title and abstract did not fully reveal the scope of the study, the full article was examined to fully assess the entire information for that specific particular. Furthermore, some grey literature was considered for this study. Google and organization websites such as United Nations Development Programme (UNDP), World Health Organization (WHO), United Nations (UN), and United Nations Office for Outer Space Affairs (UNOOSA) were used to get the most up-to-date information. PICO was used as the strategy to undertake the systematic review.

**Figure 1:**
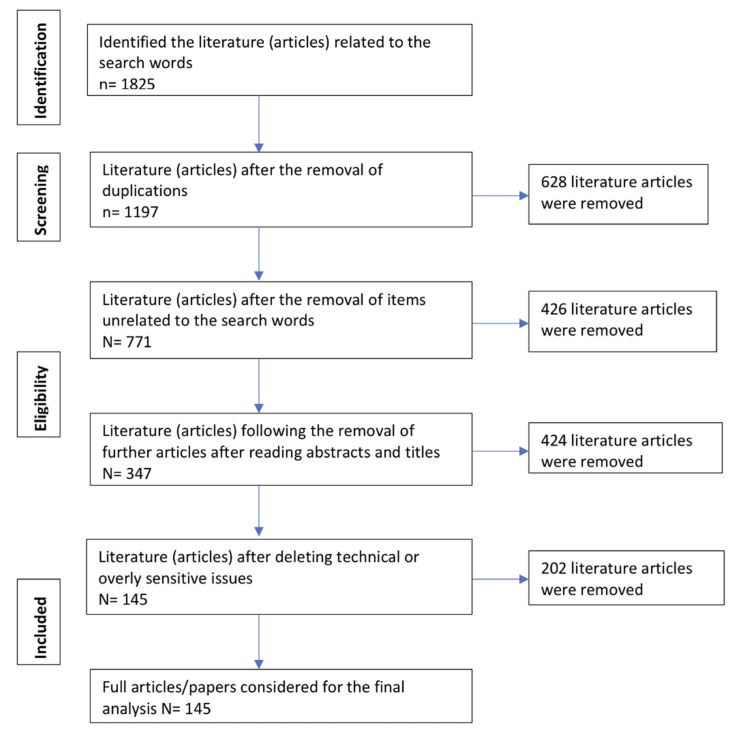
Systematic review—PRISMA model flow diagram (inclusion and exclusion criteria).

Based on Google, Google Scholar, PubMed, Science Direct, Web of Science and Scopus, a total of 1825 relevant articles were identified. However, more than 600 (628) duplicates were identified and these were deleted. Moreover, literature that was not closely related to the search words led to the deletion of a further 426 articles. At this stage, 771 articles had been considered for assessment. After careful consideration of the titles and abstracts, a further 424 articles were removed. At this stage, 347 articles had been considered for the analysis. After reading these 347 papers, a further 202 articles were deleted, as they had either highly technical or overly sensitive issues. Finally, 145 papers were considered for the study. Of these 145 papers, Table 2 presents the top 10 papers, which are the most relevant and highly cited articles. Figure 1 provides information on the inclusion and exclusion criteria of the literature.

## 3. Review Results

### 3.1. Pandemics and Their Impact on Various Population Groups

Pandemics/disasters often leave a significant impact on human health and development. This includes, but is not limited to, loss of human lives, livelihood issues, and psycho-social problems. Pandemics can create long-term imbalances in societies and communities. The challenges confronted by the general public due to the pandemic have revealed inadequacies in the areas of managing health risks, injuries, diseases, disabilities, psychological problems and deaths [18]. The COVID-19 pandemic has affected all aspects of human life and the global economy [19]. The World Trade Organization (WTO) and Organization for Economic Cooperation and Development (OECD) marked the COVID-19 pandemic as the greatest peril to the world economy since the financial emergency of 2008–2009 [20]. Emerging issues related to jobs and income of millions of people, social safety net, future of income support schemes, the burden on women, and the plight of migrants and informal sector workers are some of the main challenges that the world is confronting [21]. Oxfam predicts that the economic crisis due to COVID-19 could push half a billion people into poverty [22]. Due to the lockdown, economic activities and livelihoods were affected in many ways, especially in the fields such as production and distribution, consumption, restriction on trade and business, large-scale uncertainties in the market, lack of access to the resources and sudden disappearance of the more informal sectors of employment/sector [23]. The global outbreak has resulted in developmental impacts on health, education, gender, economy, politics and the environment. The COVID-19 pandemic has exposed huge health inequalities across countries and within countries due to existing social stratification and resource sharing. People from lower socio-economic strata lack access to essential healthcare services during the pandemic time [24]. The economic decline during the pandemic has significantly affected people from the lower socio-economic stratum [23]. This pandemic has marked a significant impact on the lives of many vulnerable sections of society, including women and children. Across countries, the number of cases related to domestic violence has increased [25]. The pandemic has had an extensive impact on the education sector [26,27,28], and all educational institutions have been closed for several months, especially in countries where vaccination proceeded at a slower pace. The pandemic has forced a worldwide lockdown, with a huge number of citizens confined to their homes [29], often resulting in social isolation. Social isolation has led to chronic loneliness and boredom, which has affected mental health, human happiness and wellbeing [25].

The pandemic affected political systems across the globe, causing ideological differences, lack of need-based initiatives, geopolitical cooperation/dysfunctions, misinformation and misleading/false claims. The COVID-19 pandemic has affected religion in many ways, including cutting short pilgrimages and journeys related to religious practices and festivities [30]. People working in the informal sector, including migrant workers, are at a high risk of poverty as their income and livelihood options are limited [31,32]. Vulnerable populations have struggled to cope with the magnitude of problems and the incidence of suicide has increased due to loss of income, livelihood and other factors [33]. Challenges of immunization, nutrition, poverty, hunger, acute undernourishment, and health inequalities, especially amongst vulnerable groups, have posed severe health and economic challenges [31].

The pandemic’s impact on social life, the economy and the financial sector has led millions of people to face an unprecedented situation related to poverty, wherein an average of 3.3 billion of the global workforce are at risk of losing their livelihoods [12,34]. Breadwinners working in the informal economy, particularly marginalized populations in low-income countries, which includes small-scale farmers and indigenous peoples, have been drastically affected [35]. According to a WHO survey, in May 2020, it was found that in 155 countries, the pandemic had severely curtailed people’s ability to avail treatment services for Non-Communicable Diseases (NCDs). This situation is of significant concern because people living with non-communicable diseases tend to be at higher risk of severe COVID-19-related illness and death [36]. While the health systems of various countries are being challenged by the increasing demand for care of COVID-19 patients, it is imperative to maintain preventive and curative health care services, especially for the most vulnerable populations, such as children, women, older persons, people living with chronic conditions, minorities and people living with disabilities [37]. The pandemic has deepened pre-existing inequalities in social, political and economic systems, including access to health services and social protection. Women with care responsibilities, informal workers, low-income families and young people have been most adversely affected by the pandemic. There has also been a significant rise in domestic violence [38]. An increase in violence against women has resulted in a threat to public health and women’s health across the globe. The health impacts of violence, particularly intimate partner or domestic violence, on women and children have significantly increased in various societies. Women who have been displaced, are refugees, and are living in conflict-affected areas are the most vulnerable [39]. Lack of education and economic insecurity has also increased the risk of gender-based violence. Without sufficient economic resources, women cannot escape from abusive partners and hence face a greater threat of sexual exploitation and trafficking [40]. Pandemic-induced poverty has also widened the gender poverty gap, pushing women into extreme poverty, as they earn less and hold less secure jobs than men [22,41]. The economic fallout for women has increased due to more unpaid care work, thereby compelling them to go back to traditional gender roles of more household and care workers [42].

Children are affected due to the pandemic and this is most visible in their health and education in various ways [43]. Children from marginalized sections have been the victims as inequalities in the teaching-learning system widened. Data show that 463 million children did not have access to the internet or digital devices for remote learning during the closure of schools [44]. Closures of schools have severely affected those children who rely on school-based nutrition programmes for their food and survival. Children suffering violence at home, refugee children, migrant children and children affected by conflict face appalling human rights violations and threats to their safety and well-being [45]. The additional stress and stigma that befall families struggling to cope have also impacted their children [45]. In the last two decades, there has been significant progress in the fight against child labour; however, the pandemic could significantly reverse this otherwise positive trend [46]. This reversal is because the crisis has enormously disrupted global education, and the lack of distance-learning solutions in many of the developing and underdeveloped countries has excluded children from online education for a very long duration. Furthermore, this trend has the potential to push millions of children into child labour [47]. Whilst the adverse socio-economic and financial impacts have fallen on the majority of households globally, there is significant inequality with some children impacted more severely, for example marginalized minority groups, disabled, street-connected and homeless populations, single or child-headed households, migrants, refugees, internally displaced persons, or people from conflict or disaster-affected areas, will be more vulnerable to child labour [48].

Beyond poverty and informality, the most explicit references to other vulnerable people and groups include older persons and people living with disabilities [49]. As the world struggles with an incomparable health crisis, older persons have become the topmost victims. The pandemic affected persons of all ages, yet older persons and those with underlying medical conditions tend to be at a higher risk of serious illness and death due to COVID-19 [50]. In the face of a life-threatening pandemic, especially during the first wave, many of the older persons faced challenges in accessing medical treatments and health care services for non-COVID ailments and chronic diseases. In developing countries, the prolonged lockdowns, weak health systems and healthcare facilities requiring out-of-pocket expenditure left millions of older people, especially those in the poorest groups, without access to basic health care, which ultimately increased their vulnerability to COVID-19 as well [51]. While older people often have been invisible in humanitarian action, the pandemic uncovered their exclusion. Older persons usually had to rely on multiple income sources, including paid work, savings, financial support from families and pensions. Additionally, for those older people living alone, isolation combined with other factors such as limited mobility creates greater risks [52]. Individuals living with disabilities represent 15% of the population [53], and their barriers related to accessing mobility, access to health services and appropriate communication have increased tremendously, which further increases their vulnerability [54]. The physical, social, economic and health impacts of COVID 19 on people with disabilities require empirical studies so that severity can be assessed and appropriate policies can be developed [55].

### 3.2. Governance Issues

The pandemic also put to test the efficiency and quality of governance and the political will of the leadership in each country. During a public health crisis, people naturally depend on their governments for security and support [56]. COVID-19 brought in a unique set of challenges to governments across the globe, such as a lack of post-crisis reconstruction and recovery, weak legal and institutional mechanisms, weak infrastructural facilities, including communication networks, a lack of systematic, periodic assessment and accounting of potential losses, and poorly managed financial, technical and human resources [57]. Spontaneous behavioural reactions such as generalized panic and rumours regarding the spread of COVID-19 were reported from across the countries and each country dealt with it using different levels of efficiency and effectiveness [58]. For example, in India, the most troubling aspect was the shortage of proper provision of safety nets (e.g., food safety) during the lockdown for the weakest and vulnerable sections of the population, which was tackled by providing free food grains and cash transfer support for three months [59]. The unprecedented pandemic situation has shown the inadequacies in the global governance structure [31]. Moreover, the spread of fake news and misinformation was a major unresolved challenge for many of the democratic governments [60].

### 3.3. Strategies for Solving Multiple, Interconnected Problems of COVID-19

The WHO report on global surveillance for human infection with novel coronavirus highlights the importance of research studies to understand the viral transmission from animals and animal handlers, which will serve as evidence to prevent outbreaks similar to COVID 19 in the future [61]. To effectively respond to a public health emergency, the health system of the country must engage and step up preparedness activities with active involvement and leadership of the health department/ministry. Public health systems play a crucial role in planning health responses to respond and recover from the threats and emergencies introduced by pandemics. In various countries, fragmentation of health services has led to limited timely interventions and responses to health crises, which shows the need to have a strong coordination mechanism in place [62]. Public health emergency preparedness requires planning and intervention activities to prevent the spread of the virus, protect against other diseases and environmental hazards, promote and encourage health-seeking behaviours, respond to the crisis, assist communities in recovery, ensure quality and accessibility of the essential health services. Highly active surveillance is needed in all countries using the WHO-recommended surveillance case definition [63]. Furthermore, epidemiologic and surveillance activities would enable the public health systems to choose the most efficient ways to control the pandemic [64]. Non-pharmaceutical interventions based on supported physical distancing have a strong potential to lower the epidemic peak [65]. Priority should be accorded to certain areas, including assessment of the global health landscape; to accepting and recognizing epidemiological, environmental and economic crisis; to ensuring health regulations, such as tobacco control; to upgrading healthcare service delivery systems; and to ensuring innovative infection control, global research collaboration, universal health coverage, and public health surveillance. To support contact tracing, governments must consider expanding the use of information technology and digital initiatives to find high-risk areas [66].

The role of effective public health surveillance is crucial both in the short term and long term because the disease may remain in isolated pockets and regions even if it ceases to be a pandemic anymore. Surveillance informs about reality on the ground and provides insights for policymakers, which is essential [67]. Exploring and using web-based open tools to modernize data reporting can help provide newer, faster insights about COVID-19 controls [68]. COVID-19 surveillance in low/middle-income countries for a longer period is a real challenge due to a lack of resources, expertise, skills, people’s attitude to tackling these issues technology transfer, financial assistance and capacity-building support is to be ensured [69].

The disease load of the pandemic is inequitably distributed among vulnerable populations [70]. People living in low- and middle-income countries have reduced capacity for self-protection (due to poor housing, sanitation and living conditions) [71] a high risk of food insecurity [72], a widened gap in health care access [73], loss of livelihoods, and a decrease in dietary intake and health care consumption [74]. Public policy needs to reorient federal, state and local governments to handle health equity issues sensibly [75]. The relevance of integrating public health efforts with broader public policy and acknowledging the role of social determinants of health is important [76]. Developing universal schemes for food assurance, minimum incomes, reforming unemployment insurance, and investment in community development will help to address health-inequity-related issues in the post-pandemic era [77].

COVID-19 is unlikely to be controlled or eliminated until there is global coverage of the population with effective vaccination. Vaccine development itself is not adequate; its mass production, affordability, global availability and acceptability in local communities are also important [78]. Strategies are needed to ensure affordability by handling Intellectual Property Rights issues and increasing production [79]. Long-term massive investment in the vaccination is needed; however, if the regular health budget is diverted for this, it will lead to long-term adverse consequences for general health indicators and development [80]. Increasing government revenue and getting grants and aid from donors and international loan providers are important [81]. Uneven distribution of vaccination is always a major challenge [82]; hence, vaccines should be distributed in stages, giving priority to older persons, high-risk individuals and people with co-morbidities [83]. The distribution must adhere to the WHO framework for allocating COVID-19 vaccines internationally based on need [84]. Vaccine hesitancy is prevalent in low-income and high-income countries alike, with sceptics found in all socioeconomic, religious and ethnic groups [85]. Culturally tailored health communication measures [86], community engagement [87] and a robust pharmacovigilance system [88] are important strategies for addressing vaccine hesitancy.

### 3.4. Role of Multi-Stakeholders in Controlling the Pandemic and Promoting the Development

COVID-19 presents a set of significant challenges to health care providers worldwide [89]. Given the complexity of the problem and the requirement of inter-sectoral collaboration, formal multidisciplinary working groups are recommended to offer relevant, effective and pragmatic solutions [90]. The pandemic is a complex phenomenon, with multiple determinants and impacts across all spheres of life. The pandemic experience serves as evidence for the need to adopt a comprehensive trans-disciplinary approach, including several experts, not only from medical sciences but also from engineering, political science, economics, humanities, psycho-social and demographic disciplines [91], as well as media that raises public awareness about health promotion and prevention [92]. The care of patients with COVID-19 can be optimized by collaborating with various multi-stakeholders to meet the demands that are required to combat the deadly disease. Multiple stakeholder engagement is critical to address the public health crises resulting from the pandemic, including but not limited to: aid donors [93,94], international aid networks, legislative and regulatory arms of the state, logistics organizations, private health care sectors [95,96], direct suppliers, media, social media [97,98,99], local aid networks, private insurance companies [100], military and para-military forces [101], government and inter-government organizations. Inputs of experts from the field of management, economics, environmental health, disaster management and other specialized disciplines to be incorporated in policy formulation based on inter-sectoral collaboration, which in turn can create programs and policies that are more efficient and feasible [90]. The support of patients, healthcare professionals and the wider community in addition to the government is equally important to address this health crisis [60].

### 3.5. COVID-19 and Social Development

The innovative, collaborative and strategic directions proposed to control the pandemic by slowing down transmission and reducing mortality associated with the pandemic are presented in Table 3.

## 4. Future Research: Moving beyond the Transdisciplinary Framework and Study Limitations

Trans-disciplinary health science research must be the prime approach to develop a universal response to COVID-19. Long-term research priorities must serve towards an evidence base for the public health system to plan or respond to future pandemics and to develop effective systems to reach out to the public [143]. The COVID-19 pandemic has been developed as a public health and developmental crisis for all countries, and this has revealed new challenges to the research community across the globe. Extensive research is needed to understand the COVID-19 crisis life cycle and its causes and consequences *(Recovery, Mitigation, Response and Preparation).* Revisiting datasets, redefining relevant methodologies, facilitating access to online resources and exploring culturally relevant approaches is critical at this juncture. The search for relevant information sources and trying to compile proper data of active as well as closed COVID-19 cases is an important task for health researchers. Research studies are needed to explore the interconnection of climate change to the development of the virus and to understand the possible environmental factors that could influence virus diffusion [144]. Comprehensive scientific studies needed to be initiated to explore COVID-19′s impact on human development, human happiness, the well-being of helping professionals, their families and others in the community. Synthesizing evidence more rapidly will help contribute towards provision of broad-ranging intervention guidelines and longer-term strategies for human happiness and well-being and social and economic recovery. Ensuring adequate quality research work, communicating thereof with multi-stakeholders and developing policy briefs for appropriate government action is a priority area. There is also a need to strengthen community-based crisis risk management, learn from the field with empirical evidence and replicate best practices. Transdisciplinary research is best suited to explore the new parameters that could be appropriate to explain COVID-19′s initial diffusion and its development as a pandemic [144].

## 5. Recommendations

The widespread prevalence of the infection and high causalities has made pandemic policies a high priority. As a response to control the pandemic, the WHO has recommended countries to develop preparatory policies to fight against the pandemic as well as address pandemic-induced developmental problems [145]. Developing appropriate COVID-19 control policies is a huge public health concern for all countries, and this requires combined inter-sectoral collaboration and government agreements through various coalitions [90]. The policy response should be two-fold: address present critical health and livelihood issues and suggest an approach to deal with the long-term issues the pandemic has introduced. The public health sector must take the lead for the whole of society, with a welfare approach to minimize the negative impacts of COVID-19 and help people restore the balance in their lives and livelihoods. This includes responding with appropriate public health emergency actions, identifying economic impacts, identifying and dealing effectively with misinformation spread about the disease [146]. Governments need to focus on providing authoritative information via multiple sources to ensure accurate data and appropriate social behaviour. Increasing transparency, ensuring proper restrictions, designing suitable prioritization guidelines about how to allocate scarce resources and making use of effective technologies are important [146]. To recognize the potential of psychological burnout from long hours of work and potential demoralization from persistent stress among health care workers is also an area that needs the urgent attention of policy framers. Vaccine and therapeutic investment, as well as research and development on COVID-19 control/elimination, is another key area. Governments need to strike a balance between protecting health and respecting human rights [146]. Identifying a new set of priorities and reworking national spending priorities will help to utilise available resources most efficiently and facilitate the return of normality in people’s lives. Governments should address the long-standing challenges of health and nutrition of low-income households, strengthen food supply chains and empower women in food chains [147]. In response to the COVID-19 crisis, the International Labour Organization (ILO) has structured the four-pillar policy framework based on international labour standards to tackle the socio-economic crisis, stimulate the economy and employment, protect workers in the workplace, and rely on social dialogue for solutions [148].

## 6. Conclusions

The world is facing unprecedented challenges due to COVID-19, and hence pragmatic and innovative approaches are needed for pandemic management. To contain the spread of the virus, public health surveillance needs to be strengthened, through research, capacity building and action. Inter-institutional collaborations can help in enhancing the quality of surveillance, preparedness and capacity building during public health emergencies. Working closely with inter-regional and national public health and emergency management plans will help to control virus transmission and other risk factors. Since the pandemic has profound and long-term economic and social impacts, an integrated model for sustainable development, the delivery of training courses, and strengthening institutional mechanisms are essential for sustainable recovery and restoring normality in people’s lives. The complex problems of pandemic threats have to be handled proactively by formulating innovative strategies and protocols to respond to similar outbreaks in the future. Furthermore, it is necessary to implement practical, evidence-based public policy measures and innovative approaches to deal with pandemic management, including developing strong linkages between strategic partners, alternative resource mapping strategies, a robust institutional and legal framework, and promoting health equity across economies.

## Figures and Tables

**Table 1:** Criteria for the inclusion and exclusion.

Number	Inclusion	Exclusion
1	Literature published between December 2019 and March 2022	Literature published before December 2019. For the discussion and introduction and discussions, articles that were published before December 2019 were not considered
2	Literature available on COVID-19, developed and developing (low-and middle-income countries), COVID-19 and health impacts in post-pandemic context	Literature that are related but very complicated and some sensitive topics such as political decisions for the COVID-19 lockdown and vaccine development
3	Literature related to the search words	Literature that is not associated with the search words
4	Literature with novel results without any geographical remit	Literature that is highly technical in nature, and articles with incomplete, aggressive or biased results
5	Other disasters/pandemics related to the COVID-19 pandemic	Articles which were published in predatory journals and literature unrelated to the pandemics/disasters
6	Reports from various national and international organizations were also accessed apart from several non-academic sources (example: newspaper reports/online news sites). Only articles/reviews in the English language were included in the study.	The vast scope of COVID19 literature which did not give precise and accurate information related to the search words

**Table 2:** Systematic analysis results—top 10 articles and their information.

No.	Title of the Article	Type of the Article	Article Description
1	“Social isolation in COVID-19: The impact of loneliness”	Review	Highlights the problem of loneliness due to social isolation due to COVID-19 and suggests ways to overcome loneliness
2	“Bouncing forward: a resilience approach to dealing with COVID-19 and future systemic shocks”	Review	Reviews the impact of COVID-19 on socio-economic development and suggests various policies, infrastructure, and systems to bounce back with a resilience approach. It addresses future similar issues with proactive strategies
3	“Challenges in ensuring global access to COVID-19 vaccines: production, affordability, allocation, and deployment”	Health Policy	Reveals the challenges involved in COVID-19 vaccines and suggests various policies to ensure global access to these vaccines
4	“The plight of essential workers during the COVID-19 pandemic”	Review	Identifies struggles of healthcare and essential workers situation during COVID-19
5	“Multivalue ethical framework for fair global allocation of a COVID-19 vaccine”	Ethical Framework	Analyzes the importance of global access to COVID-19 vaccines and presents an ethical framework to make sure it is globally accessible to everyone.
6	“The Great Lockdown in the Wake of COVID-19 and Its Implications: Lessons for Low and Middle-Income Countries”	Review	Reveals the impact created by the great lockdown imposed due to COVID-19 and presents lessons for low- and middle-income countries to fight against COVID-19
7	“COVID-19: Impact on the Indian economy”	Policy Document	Analyzes the impact of COVID-19 on the Indian economy and suggests various policies and recommendations for different sectors
8	“Guidelines for Responding to COVID-19 Pandemic: Best Practices, Impacts, and Future Research Directions”	Review	Based on the COVID-19 pandemic experience the study presents guidelines for the improvement of workforce-related issues, demand and supply chain, and insurance needs.
9	“Multistakeholder Participation in Disaster Management—The Case of the COVID-19 Pandemic”	Review	Presents the need, policies and strategies required to fight against the COVID-19 pandemic through multi-stakeholder participation
10	“The effect of control strategies to reduce social mixing on outcomes of the COVID-19 epidemic in Wuhan, China: a modelling study”	Review	Analyzes the effectiveness of the physical distance measures related to the COVID-19 pandemic

**Table 3:** Strategies for COVID-19 and beyond.

Strategies
Identify innovative and culturally acceptable measures to prevent similar public health crises which explores and accommodates strategies beyond conventional economic lockdowns [102,103]
Identify easily available, culturally adaptable local technology, which is easily accessible and affordable to everyone [104,105]
Ensure that the most vulnerable populations are consulted and included in planning and response [106,107]
Organise communities to ensure that essentials including alternative livelihood opportunities to cater to needs related to food, clean water, essential healthcare and other basic services [108,109,110,111,112]
Advocate and promote priority-based social welfare services and in a social policy environment that services adapt, remain open and pro-active in supporting communities and vulnerable populations particularly women, children, elderly and persons with special needs [113,114]
Facilitate easily acceptable physical distancing with social solidarity advocating for the advancement and strengthening of social welfare services as an essential protection against the disaster [115]
Identify adaptable or easily doable strategies and remain open and adapt to the conditions based on available successful examples of best practices [116,117,118]
Respond to the pandemic situation with inputs from social and behavioural sciences to develop a vision beyond this crisis and translate fear, sorrow and loss into empowerment and social transformation [119]
Ensure realistic forecast, targets and goals for prevention [120,121] and control using integrated environmental and health management perspective
Promote and ensure community participation and empowerment [122,123]
Promote behavioural modification (build ownership) [124]
Work with public-private partnership modes in research, development and health care delivery [125,126]
Ensure social participation [127], long-term commitment and leadership [128,129,130]
Use and encourage e-reporting [131,132], community-controlled partnerships and intervention [133]
Develop capabilities at all levels for handling emergencies, pandemic prevention and management [134,135]
Ensure responsible and competent state leadership which includes a women’s leadership component [136,137]
Promote greater participation and accountability of local communities and other stakeholders [138,139]
Strengthen inter-organizational coordination and local responsibility with centre’s coordination [140,141,142]

## Data Availability

Not applicable.
